# Biofilm infections of endobronchial valves in COPD patients after endoscopic lung volume reduction: a pilot study with FISHseq

**DOI:** 10.1038/s41598-024-73950-3

**Published:** 2024-10-04

**Authors:** Eva Pappe, Ralf-Harto Hübner, Jacopo Saccomanno, Hadis Darvishi Nakhl Ebrahimi, Martin Witzenrath, Alexandra Wiessner, Kurosh Sarbandi, Zhile Xiong, Laura Kursawe, Annette Moter, Judith Kikhney

**Affiliations:** 1 Department of Infectious Disease, Respiratory Medicine and Critical Care, Charité – Universitätsmedizin Berlin, corporate member of Freie Universität Berlin, Humboldt- Universität zu Berlin, Hindenburgdamm 30, 12203 Berlin, Germany; 2 German Center for Lung Research (DZL), Berlin, Germany; 3 Capnetz Foundation, Hannover, Germany; 4 Institute of Microbiology, Infectious Diseases and Immunology, Biofilmcenter, Charité – Universitätsmedizin Berlin, corporate member of Freie Universität Berlin, Humboldt- Universität zu Berlin, Hindenburgdamm 30, 12203 Berlin, Germany; 5 MoKi Analytics GmbH, Berlin, Germany; 6 Moter Diagnostics, Berlin, Germany

**Keywords:** COPD, Endoscopic lung volume reduction, Bacterial biofilms, Exacerbations, Chronic obstructive pulmonary disease, Infection

## Abstract

Endoscopic lung volume reduction (ELVR) using endobronchial valves (EBV) is a treatment option for a subset of patients with severe chronic obstructive pulmonary disease (COPD), suffering from emphysema and hyperinflation. In this pilot study, we aimed to determine the presence of bacterial biofilm infections on EBV and investigate their involvement in lack of clinical benefits, worsening symptomatology, and increased exacerbations that lead to the decision to remove EBVs. We analyzed ten COPD patients with ELVR who underwent EBV removal. Clinical data were compared to the microbiological findings from conventional EBV culture. In addition, EBV were analyzed by FISHseq, a combination of Fluorescence in situ hybridization (FISH) with PCR and sequencing, for visualization and identification of microorganisms and biofilms. All ten patients presented with clinical symptoms, including pneumonia and recurrent exacerbations. Microbiological cultures from EBV detected several microorganisms in all ten patients. FISHseq showed either mixed or monospecies colonization on the EBV, including oropharyngeal bacterial flora, *Staphylococcus aureus*, *Pseudomonas aeruginosa*, *Streptococcus* spp., and *Fusobacterium* sp. On 5/10 EBV, FISHseq visualized biofilms, on 1/10 microbial microcolonies, on 3/10 single microorganisms, and on 1/10 no microorganisms. The results of the study demonstrate the presence of biofilms on EBV for the first time and its potential involvement in increased exacerbations and clinical worsening in patients with ELVR. However, further prospective studies are needed to evaluate the clinical relevance of biofilm formation on EBV and appropriate treatment options to avoid infections in patients with ELVR.

**Supplementary Information:**

The online version contains supplementary material available at 10.1038/s41598-024-73950-3.

## Introduction

Chronic obstructive pulmonary disease (COPD) belongs to the most devastating lung diseases worldwide with an annual increase in prevalence^1^. Patients with advanced COPD suffer from severe lung emphysema, which is characterized by the destruction of alveolar septa and irreversible airflow limitation^2^. Endoscopic lung volume reduction (ELVR) with endobronchial valves (EBV) is a treatment option for a subset of patients with severe lung emphysema^3^. Randomized trials have shown improvement of lung function, quality of life and exercise capacity after ELVR^4–6^. Nevertheless, some patients do not sufficiently benefit from ELVR, and the EBVs must be removed. Most frequent indications for EBV removal are recurrent exacerbations or pneumonia^7^.

Acute COPD exacerbations are defined as acute episodes of worsening of symptoms^8^. Pathophysiologically, this is based on an acute inflammation of the upper and lower respiratory tract^9^. Recurrent exacerbations are associated with worse quality of life, deterioration of lung function and increased mortality^10–12^. Several risk factors and triggers have been identified, among which respiratory viral and bacterial infections are a predominant cause^13^. Approximately half of the respiratory infections are caused by bacterial infection with *Haemophilus influenzae*, *Moraxella catarrhalis*,* Streptococcus pneumoniae*, and/or *Pseudomonas aeruginosa* as the most common isolates from standard microbiological culture^14^. However, these bacteria are also often detected in COPD patients during stable episodes, colonizing the airways^15^. The basis for this is thought to be the formation of mucus plugs in the remodeled airways, which provides a nutrient-rich substrate for the colonizing bacteria^16^. Studies have shown that these conditions were associated with increased airway inflammation and exacerbation rates^14,15^.

Among patients with medical devices, device-related infections represent a serious complication that is often caused by the formation of bacterial biofilms on the surface of the devices. Biofilms are composed of single or multiple species of microorganisms embedded in a matrix of extracellular polymers^17^. Biofilms are characterized by tolerance towards antimicrobial agents and host immune defenses, resulting in recurrent infections^17,18^. Moreover, diagnosis of biofilms with conventional microbiological methods is challenging, as microbiological culture may remain negative for the bacteria that form the biofilm. Currently, the only method for direct detection of biofilm-associated infections on medical devices, is FISHseq, the combination of Fluorescence in situ hybridization (FISH) with PCR and sequencing, – a method that combines molecular detection of microorganisms with in situ visualization by fluorescence microscopy. FISHseq delivers information about the identity, formation and localization of microorganisms in/on the specimen (tissue/implant), which helps to distinguish between contamination and infection^19^.

So far, little is known about biofilm infections in patients who underwent ELVR. Existing evidence suggests that COPD patients seem to have more post-procedural exacerbations after ELVR, if bacterial colonization of the lower airways is present^20^. However, studies addressing the presence of biofilms on EBVs are lacking. In this pilot study, we aimed to determine whether biofilm infections are present on EBV using the advanced molecular microscopic method FISHseq. Furthermore, we investigated the involvement of bacterial biofilms in the lack of clinical benefits, worsening symptomatology, and increased exacerbations, which could lead to the decision to remove EBVs.

## Methods

### Study population

We conducted a pilot study with 10 COPD patients, who underwent ELVR with implantation of EBVs and were followed-up at Charité – Universitätsmedizin Berlin within Lungenemphysemregister e.V. (LE-Registry). The LE-Registry is a national, multicenter, non-profit, manufacturer-independent registry (lungenemphysemregister.de) that collects clinical data exclusively from patients with severe lung emphysema (GOLD III and IV) in Germany^21^. It is registered at the German Clinical Trials Register (DRKS00021207). Inclusion and exclusion criteria were as described before^21^.

### Ethics approval and consent to participate

The studies involving human participants were reviewed and approved by the Institutional Ethics Committee of Charité – Universitätsmedizin Berlin (EA2/149/17). The patients provided their written informed consent to participate in this study. All identifiable information was anonymized to protect the privacy of the participants The study was executed in accordance with ethical standards outlined in the Helsinki Declaration.

### Sampling

Microbiological cultures and EBV samples were obtained during flexible bronchoscopy. Patients were placed in the supine position and nasal oxygen was administered, depending on the patients’ needs to maintain a peripheral oxygen saturation > 92%. Intravenous sedation was induced with 2.5 mg midazolam and maintained with propofol boluses. To secure the airway, patients were intubated with a 7.5 mm endotracheal tube (Bronchoflex. Rüsch GmbH, Germany), while breathing spontaneously during flexible bronchoscopy. The bronchoscope was introduced orally and advanced to target subsegmental bronchi. A trap for bronchial washing fluid was connected to the suction channel after passage of the vocal cords. Neither topical lidocaine nor suction was used until the vocal cords were passed. By using the flexible bronchoscope 5-10 ml of sterile saline was instilled into the subsegmentary bronchus before the EBV and aspirated. The bronchial washing fluid was subsequently transported to a laboratory (Labor Berlin – Charité Vivantes GmbH) for routine microbiological culture.

The EBV were removed using endobronchial forceps and immediately placed into FISHopt^®^ FISH fixation solution (MoKi Analytics, Berlin, Germany) and subsequently transported to a laboratory (Moter Diagnostics/MoKi Analytics GmbH) for FISHseq.

### Microbiological culture

Bronchial washing fluids were submitted for standard microbiological culture. Fluid samples were obtained during flexible bronchoscopy before and after ELVR, as well as according to clinical estimation. Cultures were incubated for at least 48 h at 37 °C in aerobic and anaerobic conditions. After 24 h, the first evaluation took place, and after 48 h the final evaluation. In case of positive culture, microorganisms were identified by an automated biochemical system and later MALDI-TOF mass spectrometry (Vitek 2 and Vitek MS, bioMérieux, Nürtingen, Germany).

### FISHseq

For FISHseq, the combination of FISH with PCR and sequencing, EBV specimens were processed as described before^22–24^. Briefly, samples were fixed, embedded in cold polymerizing resin, sectioned, and submitted to hybridization. After incubation for 1.5 h in a dark humid chamber at 50°C, slides were rinsed with water, air-dried, and mounted for microscopy with an epifluorescence microscope (Axio Imager.Z2; Carl Zeiss, Jena, Germany) equipped with narrow band filter sets (AHF analysentechnik, Tübingen, Germany). Sections were first screened with the pan-bacterial, 16S rRNA directed probe EUB338 ^25^ and the nonsense probe (NON338) to exclude unspecific probe binding^26^. To detect fungi in the samples, we useda eukaryotic, 18S rRNA-directed probe EUK516^25^ to detect *Eukarya* for particular yeasts and the *Candida* specific, 18S rRNA-directed probe CAND10^27^. The nucleic acid stain DAPI (4’,6-diamidino-2-phenylindole) was applied as counterstain to visualize host cell nuclei and microorganisms that contain no or too few ribosomes to be visualized by microscopy. A fourth microscopic channel was left without fluorochrome to control autofluorescence of the device material. Upon detection of microorganisms, a panel of FISH probes was applied for identification on a genus- or species-specific level^28^. Each hybridization experiment was controlled using positive reference strains and negative control strains with minimum mismatches in the probe target sequence^29^. FISH targets the ribosomes, which are highly abundant in replicating and metabolically active microorganisms. Therefore, FISH-positive microorganisms can be regarded as active, whereas FISH-negative microorganisms that did not contain enough ribosomes to elicit a FISH signal, can be classified as inactive^30,31^. Sample sections consecutive to those used for FISH were submitted to DNA extraction, PCR amplification and sequencing of part of the 16 S rRNA-gene using pan-bacterial primers as described^23^. Sequences were analyzed using the diagnostic-grade Centroid database from SmartGene (SmartGene Services SARL, Lausanne, Switzerland)^32^.

### Data collection

Medical records were analyzed retrospectively for each patient. The baseline characteristics were recorded, as well as dates of EBV implantation and explantation, lung function data at 3-month follow-up, clinical symptom burden, inflammatory markers (including WBC and CRP), frequency of exacerbations, results of microbiological cultures and radiological findings.

## Results

### Baseline characteristics

We included 10 patients with COPD (GOLD III or IV), who underwent EBV removal after ELVR. Detailed baseline characteristics are shown in Table 1. The patient population consisted predominantly of females (7/10), with a mean age of 63 ± 6.9 years and a significant smoking history (52 ± 16.7 pack years). The majority of patients were classified as GOLD IV (6/10). Baseline lung function tests revealed substantially reduced FEV1 (Mean 28.2 ± 7.9% predicted) and diffusion capacity (DLCO, Mean 29.1 ± 11.8% predicted), alongside high residual volumes (RV, Mean 235.6 ± 45.3% predicted). The baseline 6-Minute Walk Distance (6MWD) was notably low, averaging 218.1 ± 114.4 m. A high symptom burden was detected by the COPD Assessment Test (CAT), St. George’s Respiratory Questionnaire (SGRQ), and Modified British Medical Research Council (mMRC) scores. Inflammatory markers at the time of EBV implantation showed a slight elevation.

**Table 1:** Patients‘ baseline characteristics before endoscopic lung volume reduction.

	Incidence
Age ± SD (y)​	63.0 ± 6.9
Male n (%)​	3/10 ​(30)
Female n (%)​	7/10 ​(70)
Pack Years M ± SD​	52.0​ ± 6.7
GOLD Classification n	
GOLD I​	0/10
GOLD II​	0/10​
GOLD III​	4/10
GOLD IV​	6​/10
Lung Function Test at Baseline​	
FEV1 (%) pred. M ± SD​	28.2 ± 7.9
RV (%) pred. M ± SD​	235.6 ± 45.3
DLCO (%) pred. M ± SD​	29.1​ ± 1.8
6-MWD (m) M ± SD​	218.1 ± ​14.4
CAT Score (points) M ± SD​	28.3​ ± 6.0
SGRQ (points) M ± SD​	70.8​ ± 7.5
mMRC-Score (points) M ± SD​	2.7​ ± 1.1
Inflammatory marker at Baseline*	
CRP (< 5 mg/L)​ M ± SD​	13.9 ± ​21.5
WBC (3.9–10.5 × 10^9^/L)​ M ± SD​	11.0 ± 2.5

FEV 1 = Forced Expiratory Pressure in 1 s; RV = Residual Volume; DLCO = Diffusion capacity; 6-MWD = 6-Minute Walk Test; CAT-Score = COPD Assessment Test; SGRQ = St. George’s Respiratory Questionnaire; mMRC-Score = Modified British Medical Research Council; CRP = C-reactive Protein; WBC = White blood; M ± SD = Mean, *Standard Deviation*; *Date of endobronchial valve implantation.

### Microbiologial culture & FISHseq

Standard microbiological culture from bronchial washing fluids detected growth of microorganisms in all patients, including several bacterial and *Candida* species (Table 2). Before EBV implantation, microorganisms such as *Escherichia coli*, *Klebsiella pneumoniae*, *Haemophilus influenzae*, *Mycobacterium scrofulaceum*, and *Aspergillus clavatus* were identified. Post-implantation cultures showed increased occurrences of *Pseudomonas aeruginosa*, *Serratia marcescens*, *Stenotrophomonas maltophilia*, *Proteus mirabili*s, and *Acinetobacter baumannii*, along with fungal species like *Candida albicans*, *Candida glabrata*, and *Aspergillus fumigatus*.

**Table 2:** FISHseq and microbiological cultures of bronchial washing fluid of patients with endobronchial valves. Bp = the length of the amplified fragment used for sequencing in base pairs (bp) from Sanger sequencing; % = percentage of the homology of the bacterial species to the sequences in the databases; * activity based on fluorescence signal intensity, which is directly correlated to microbial ribosome-content; description of the biofilm as thin (up to three bacterial layers) and mature (more than three bacterial layers).

ID	Microbiological culture of bronchial washing fluid before EBV implantation	Microbiological culture of bronchial washing fluid after EBV implantation	FISHseq result Formation of microorganisms
1	*Escherichia coli* *Klebsiella pneumoniae*	*Pseudomonas aeruginosa* *Escherichia coli* Oral flora	*Pseudomonas aeruginosa* (498 bp, 99.8%) Active* mature biofilms
2	*-*	*Serratia marcescens* *Klebsiella pneumonia* Oral flora *Candida tropicalis*	Mixed oral biofilm with *Streptococcus* spp. *Fusobacterium nucleatum* Active biofilm
3	Oral flora	*Stenotrophomonas maltophilia* Oral flora *Candida albicans/dubliniensis*	*Streptococcus parasanguinis* (394 bp, 98.6%) Thin, active biofilm
4	*Haemophilus influenzae* *Mycobacterium scrofulaceum* *Candida albicans*	*Candida albicans* Gram-positive cocci Oral flora	Highest homology to *Corynebacterium striatum* (391 bp, 91.3%) Single, active bacteria
5	Oral flora	*Aspergillus fumigatus* *Proteus mirabilis* *Acinetobacter baumannii* *Candida albicans* *Candida glabrata* Oral flora	Single active rods, partly intracellular No detection of *Candida*
6	Oral flora	*Staphylococcus aureus* *Penicillium* sp. *Serratia marcescens* C*andida albicans*	*Staphylococcus aureus* (501 bp, 99.9%) Single bacteria
7	*-*	*Klebsiella pneumoniae* *Staphylococcus aureus* *Candida albicans* Oral flora	Thick biofilms including *Staphylococcus aureus* (moderate amounts, 504 bp, 99.9%) No detection of *Candida* or *Klebsiella*
8	Oral flora	*Candida albicans* *Candida glabrata* Oral flora *Aspergillus fumigatus* *Staphylococcus aureus*	Mixed microcolonies of rods No detection of *Candida*
9	*Aspergillus clavatus*	*Aspergillus clavatus* *Penicillium* sp. *Pseudomonas aeruginosa*	Negative No detection of microorganisms
10	*Staphylococcus aureus* Oral flora	*Staphylococcus aureus* Oral flora	*Staphylococcus aureus* (499 bp, 99.9%) Thin biofilms

**Fig. 1:**
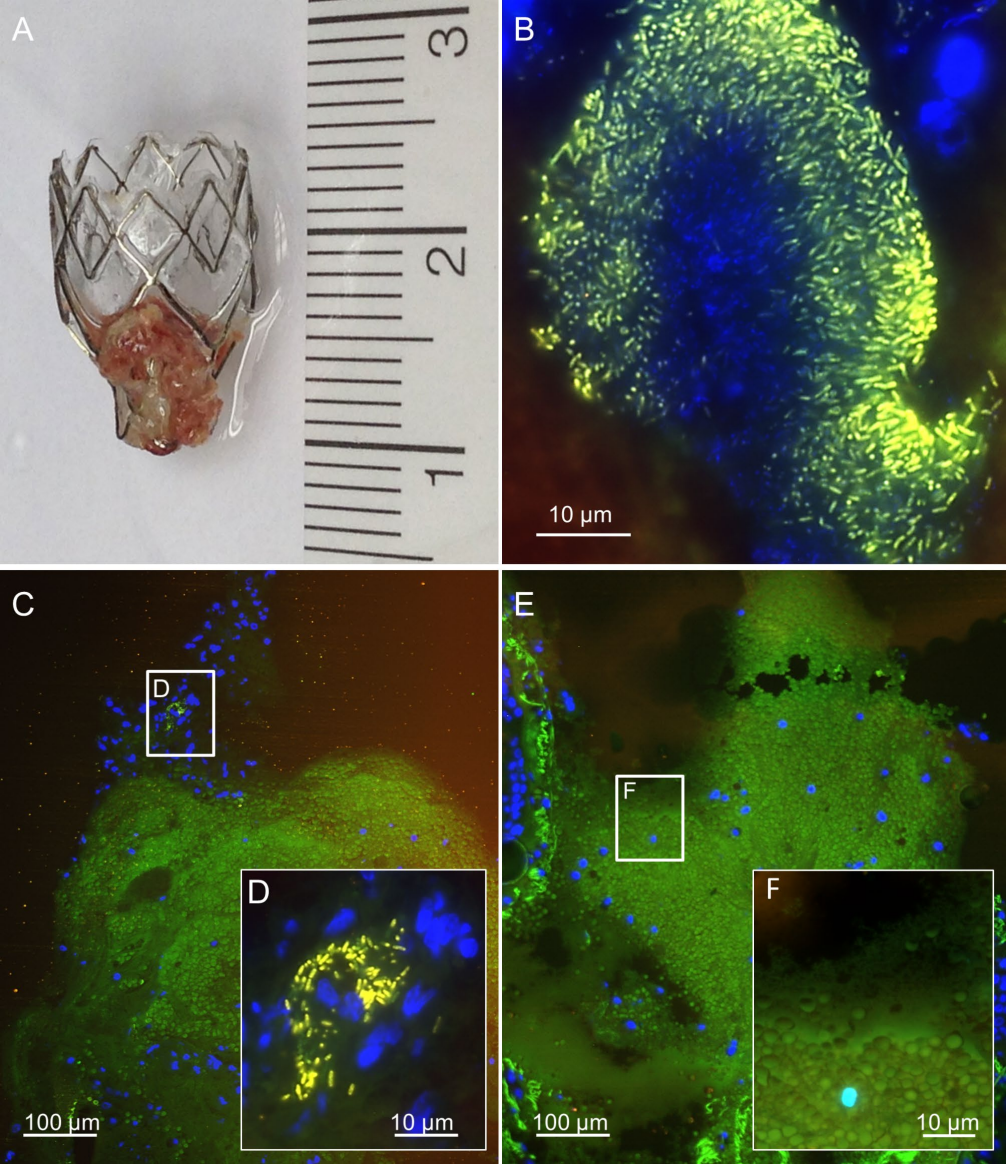
FISHseq analysis of patient 1 showing a mature *Pseudomonas aeruginosa* biofilm on endobronchial valve. Fluorescence in situ hybridization (FISH) of the endobronchial valve (EBV) from patient no. 1 fixed in the operating room. Histological sections show in green the autofluorescent tissue background, in yellow the *Pseudomonas. aeruginosa*-specific FISH probe PSMG-Cy3^46^ and in blue the nucleic acid stain 4’,6-diamidino-2-phenylindole (DAPI). (**A**) – Macroscopic impression of the explanted EBV. (**B**) – FISH visualizing rod-shaped *Pseudomonas sp.* positive for PSMG-Cy3. (**C**) – Tissue overview showing host cell nuclei in blue and the green tissue background. (**D**) – Higher magnification of the inset marked in C shows a different position with rod-shaped *Pseudomonas sp.* in PSMG-Cy3 and host cell nuclei in blue. (**E**) – Tissue overview showing host cell nuclei in blue and the green tissue background. (**F**) – Higher magnification of the inset marked in E shows autofluorescent erythrocytes and tissue background in green with host cell nuclei in blue, no bacteria are detectable.

**Fig. 2:**
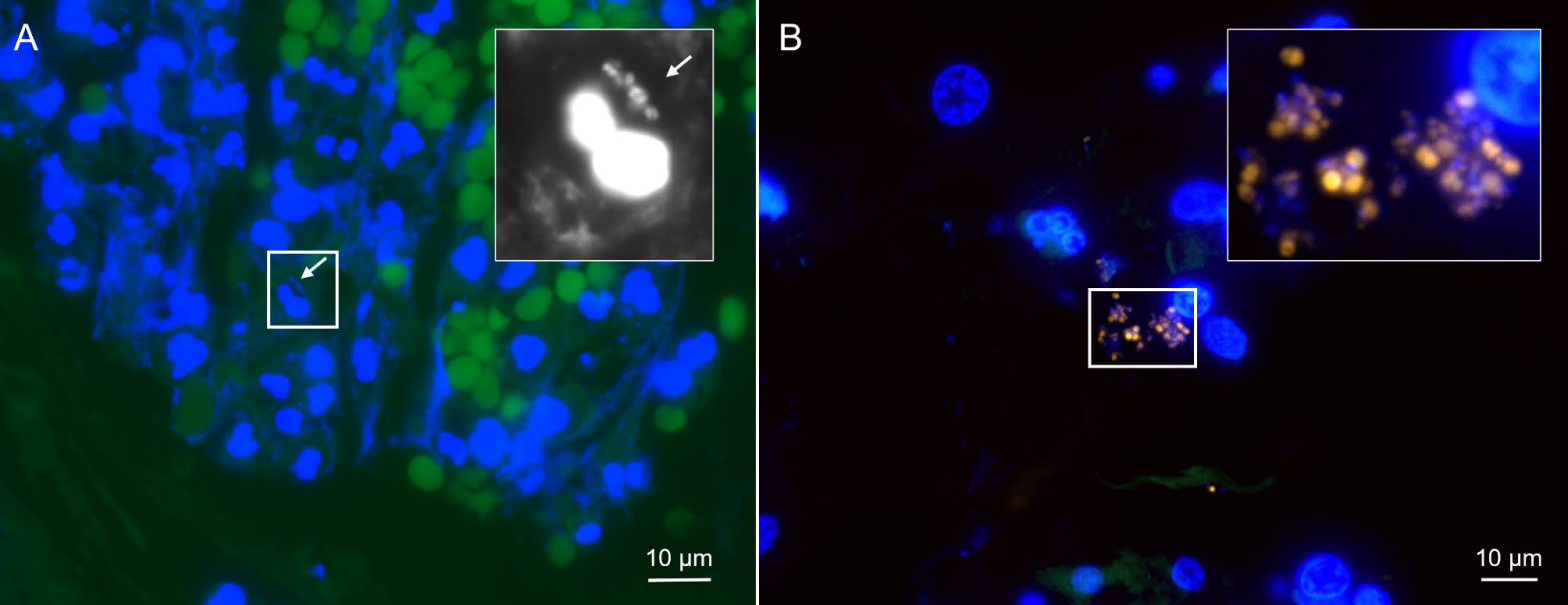
Examples of single bacteria and thin biofilms in tissue attached to the EBVs as visualized by FISH. (**A**) – Single bacteria (patient 6) and host cell nuclei shown with the nucleic acid stain DAPI (bacteria beside a host cell nucleus shown enlarged in the inset in black and white, marked with an arrow). Note the erythrocytes with strong background autofluorescence in green. (**B**) – Thin *Staphylococcus aures* biofilms (patient 10) as visualized by FISH. Shown is the overlay of the microscopic channels for the nucleic acid stain DAPI (blue), and the *Staphylococcus aures* specific FISH probe SAU in Cy3 (orange). The inset marks the region that is shown enlarged in the inset. Note the varying FISH signal intensity of bacteria due to their different ribosome content.

**Fig. 3:**
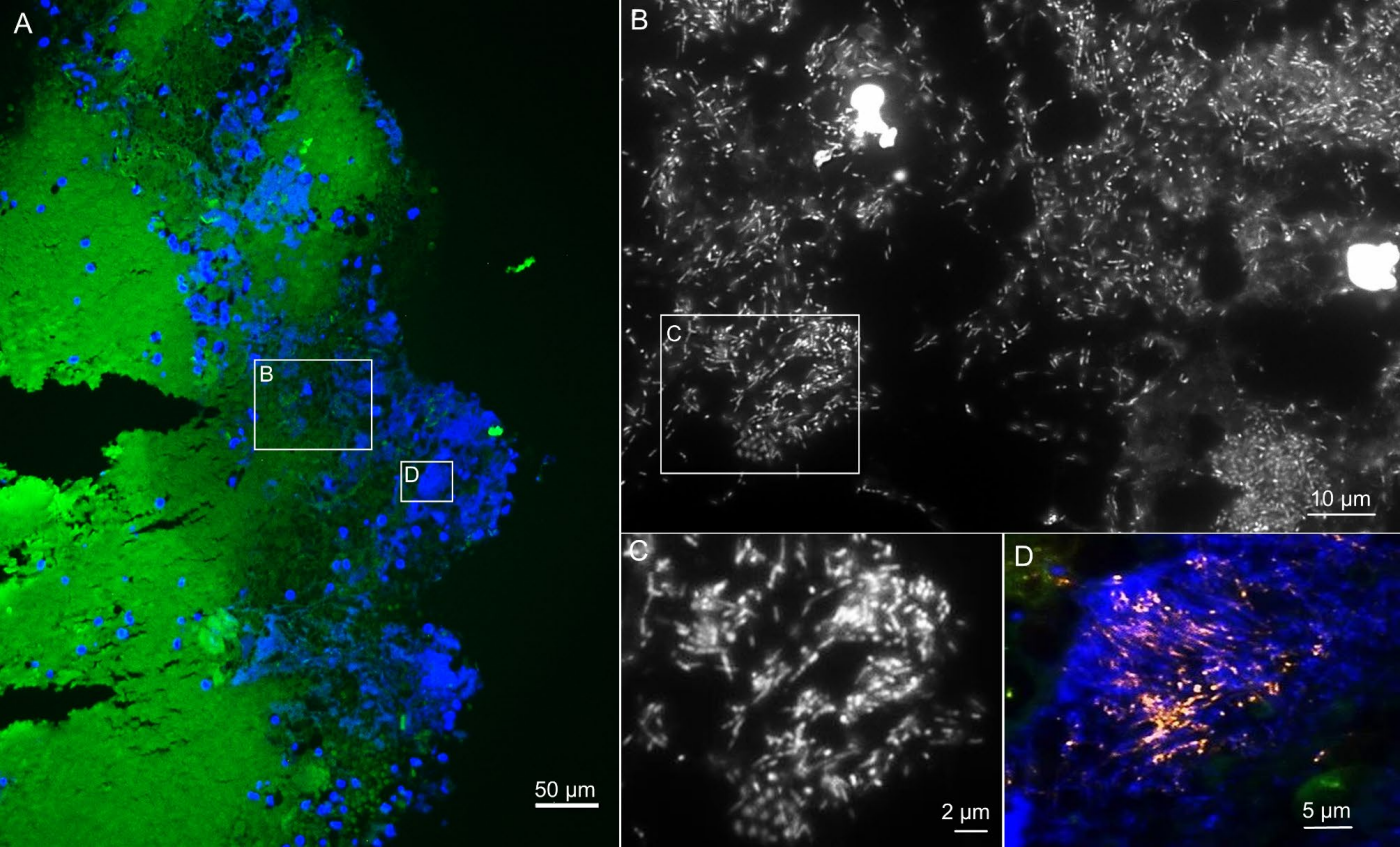
FISHseq analysis of patient 2 showing oral biofilm on EBV. Fluorescence in situ hybridization (FISH) of the endobronchial valve (EBV) from patient 2 fixated in the operating room. Histological sections show in green the autofluorescent tissue background, in orange the Fusobacterium nucleatum-specific FISH probe FUNU-Cy3 (sequence deposited in Probebase, where details on the probe are available^47^) and in blue the nucleic acid stain 4’,6-diamidino-2-phenylindole (DAPI). (**A**) – Overview of the sample showing the tissue background in green and the nucleic acid stain DAPI in blue. Extensive biofilms are visible as blue clouds. (**B**) - Higher magnification of the inset A shows that the biofilm consists of rod shaped bacteria and cocci, in line with oral flora (DAPI filter set in black and white). (**C**) – Higher magnification of the inset in B showing rods and cocci. (**D**) - Higher magnification of the inset A shows that the biofilm contains Fusobacterium nucleatum (orange). Nucleic acids in blue and tissue background in green.

Using FISHseq, no microorganisms were detected in 1/10 patients, whereas bacteria were detected in 9/10 patients (Table 2). Among those, FISHseq visualized single bacteria and microcolonies in 4/10 patients bacterial biofilms in 5/10 patients. In 4/5 patients with biofilms, FISHseq confirmed the culture results, including single species biofilms with *Pseudomonas aeruginosa* (Fig. 1), *Streptococcus parasanguinis* and *Staphylococcus aureus* (Fig. 2), and multispecies biofilms with oral flora (Fig. 3). In 1/5 patients, microbiological culture showed growth of *Stenotrophomonas maltophilia* and oral flora, while FISHseq detected an active biofilm with *S. parasanguinis*. In none of the patients with microbiological cultures positive for *Candida* FISH detected *Candida* in the valve samples.

### Clinical findings in patients with active bacterial biofilms

**Fig. 4:**
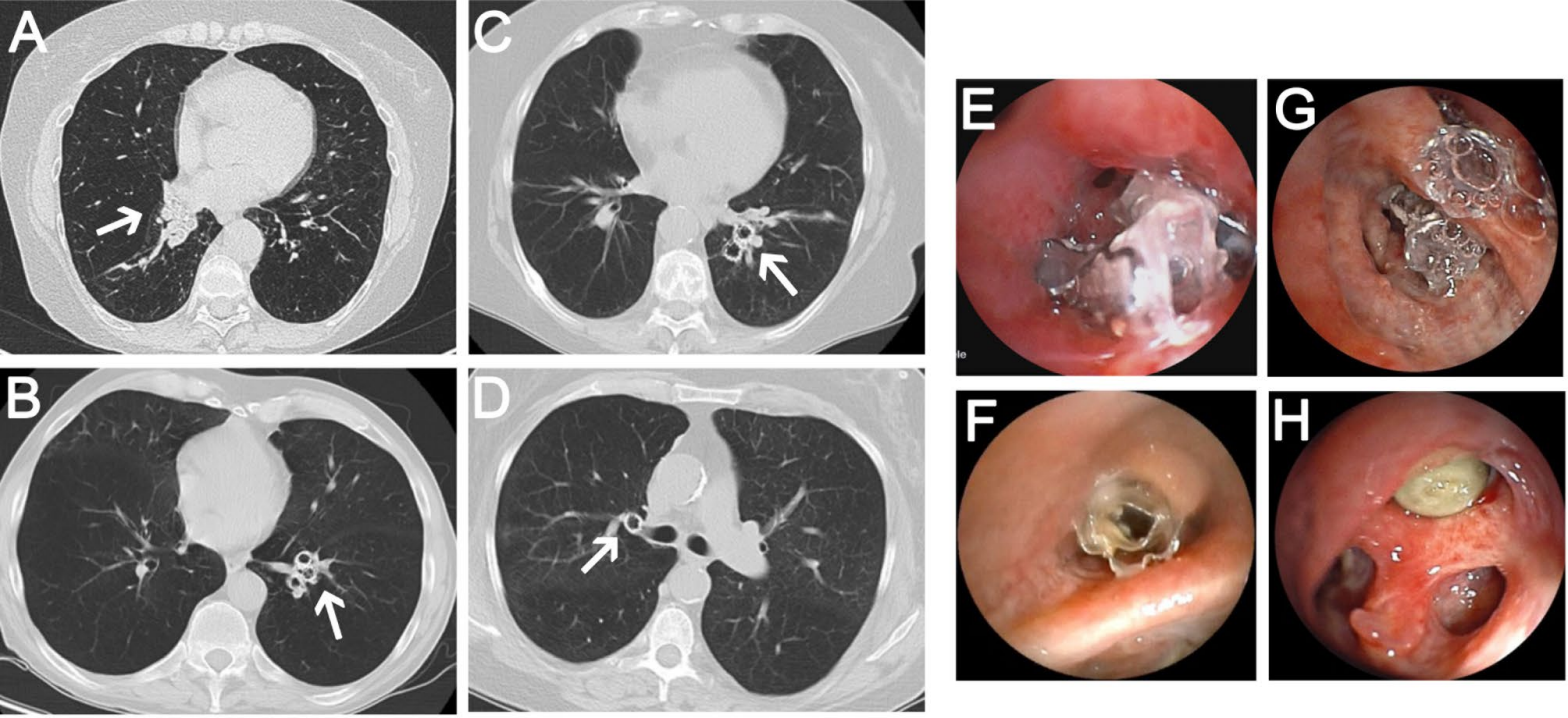
Radiological and bronchoscopic findings in patients after endoscopic lung volume reduction: (**A**) – CT scans of patient no. 1 with biofilm presented mucus layers on endoscopic valves (EBV) with almost complete obstruction of the right middle lobe. The White arrow points the mucus layer. (**B**) CT scans of patient no. 2 with biofilm showed signs of mucus layers on EBV in the left lower lobe, indicated by the white arrow. (**C**) No mucus layers were observed in CT scans of patient no. 5 and patient no. 6 (**D**), both without bacterial biofilm on EBV. (**E**-**H**) Bronchoscopy visualized mucus layers on EBV in patient no. 1, 2, 5 and 6.

At the 3-month follow-up, patients with active biofilm on EBV had lower FEV1 (24 ± 4.6 vs. 32.8 ± 8.7% pred.), higher RV (230.2 ± 91.2 vs. 206.0 ± 37.8% pred.), lower DLCO (23.5 ± 1.9 vs. 38.3 ± 12.1% pred.), and shorter 6-MWD (238.3 ± 105.6 vs. 298.8 ± 82.3 m) compared to those without biofilm (Table 3). Interestingly, symptom burden was lower in the biofilm group (CAT scores 17.0 ± 3.6 vs. 28.0 ± 3.6; SGRQ scores 43.8 ± 0.4 vs. 63.9 ± 15.5). The mMRC scores were similar (3.0 ± 0.6 vs. 3.0 ± 1.2). Atelectasis of the target lobe was observed in 2/5 of patients with active biofilm and 4/5 without. Exacerbations within six months post-EBV implantation were more frequent in the biofilm group (5/5 vs. 3/5). Radiological findings of mucus layers were more common in the biofilm group (4/5 vs. 1/5), while bronchoscopic findings of mucus layers were present in 4/5 of patients in both groups (Fig. 4). Granulation tissue development on EBV occurred in 3/5 of patients in both groups. The duration of EBV implantation until EBV removal and FISHseq analysis had a mean of 362.0 ± 352.4 days for patients with active biofilm and 317.0 ± 430.7 days for those without. Reasons for EBV removal included exacerbations or infections (4/5 with biofilm, 3/5 without), missing clinical benefit (1/5 in both groups), and pneumothoraces (0/5 with biofilm, 1/5 without). Re-implantation of EBV occurred in 3/5 of patients with biofilm and 2/5 without. Exacerbations within six months after removal were reported in 3/5 of patients with biofilm and 2/5 without.

**Table 3:** Clinical findings in patients with and without active bacterial biofilms on endobronchial valves detected with FISHseq.

	Patients with active biofilm on EBV		Patients without active biofilm on EBV	
Lung Function Test at 3-Month Follow-up				
FEV1 (%) pred. M ± SD​	24	± 4.6	32.8	± 8.7
RV (%) pred. M ± SD​	230.2	± 91.2	206.0	± 37.8
DLCO (%) pred. M ± SD​	23.5	± 1.9	38.3	± 12.1
6-MWD (m) M ± SD	238.3	± 105.6	298.8	± 82.3
CAT (points) M ± SD	17.0	± 3.6	28.0	± 3.6
SGRQ (points) M ± SD	43.8	± 0.4	63.9	± 15.5
mMRC (points) M ± SD	3.0	± 0.6	3.00	± 1.2
Atelectasis of target lobe	2/5		4/5	
Exacerbations within 6 Month after EBV Implantation (n/all patients)	5/5		3/5	
Radiology Findings of Mucus Layers after EBV Implantation	4/5		1/5	
Bronchoscopic findings of mucus layers after EBV Implantation	4/5		4/5	
Development of Granulation Tissue after EBV Implantation	3/5		3/5	
Time of EBV Removal/FISHseq Analysis (d) M ± SD	362.0	± 352.4	317.0	± 430.7
Reasons for EBV Removal n (%)​				
Exacerbations/Infections	4/5		3/5	
Missing clinical benefit	1/5		1/5	
Pneumothoraces	0/5		1/5	
Inflammatory marker at Time of EBV removal				
CRP (< 5 mg/L)​ M ± SD​	4.7	± 4.0	14.1	± 24.9
WBC (3.9–10.5 × 10^9^/L)​ M ± SD​	11.1	± 2.8	11.1	± 1.1
Re-Implantation of new EBV after removal	3/5		2/5	
Exacerbations within 6 Month after EBV Removal (n/all patients)	3/5		2/5	

FEV 1 = Forced Expiratory Pressure in 1 s; RV = Residual Volume; DLCO = Diffusion capacity;6-MWD = 6-Minute Walk Test; CAT-Score = COPD Assessment Test; SGRQ = St. George’s Respiratory Questionnaire; mMRC-Score = Modified British Medical Research Council; EBV = Endobronchial Valves; CRP = C-reactive Protein; WBC = White blood; M ± SD = Mean,* Standard Deviation*.

## Discussion

Although ELVR is assumed to be a safe method with rarely occurring long-term complications after implantation, exacerbations or pneumonia are frequent reasons for EBV removal^7^. Previous studies have shown that patients with colonized airways tend to have significantly more exacerbation after ELVR^20^ and suggested that foreign bodies in the airways can be colonized by microorganisms causing recurrent pulmonary infections^33,34^. For medical devices in different fields, including cardiovascular, urogenital or dental devices, biofilm formation has been described on the device surfaces, leading to diagnostic and therapeutic challenges^19,24,35^.

In clinical practice, it is often difficult to decide, if EBV removal is required, because of recurrent exacerbations despite successful lung volume reduction, or, after EBV explantation, if the patient is suitable for renewed ELVR. Therefore, in this pilot study, we investigated the presence of biofilm infections on explanted EBVs using FISHseq and examined the involvement in the lack of clinical benefits, symptom worsening, and increased exacerbations leading to the decision of EBV removal.

All 10 COPD patients investigated in this pilot study showed growth of microorganisms in standard microbiological culture, whereas FISHseq visualized bacteria in 9/10 patients. The microorganisms identified by standard microbiological cultures corresponded mostly to the FISHseq findings, with FISHseq visualizing the key bacteria on EBV out of several microorganisms detected by culture. The patient who was FISHseq negative had the EBV for only six days before removal.

FISHseq did not find *Candida*, whereas culture grew fungi in almost all samples. This can be explained by a larger sampling area from the washing fluid/or contamination by oral flora, in particular, since no relevant fungal biofilms were found. In our study, bronchoscopic procedures were performed via the mouth, a standard approach that can introduce contamination from oral flora, including *Candida* species. *Candida* are common in the oral cavity and upper respiratory tract, so their presence in samples likely reflects oropharyngeal contamination rather than actual endobronchial colonization or infection.

With this study, we were able to demonstrate biofilms on EBV for the first time. Out of nine patients with positive FISHseq findings, biofilms on EBV were present in five patients. FISHseq visualized oral flora (Fig. 3) as well as mono- and multispecies bacterial communities on EBV, with the most prominent species being *P. aeruginosa* (Fig. 1) or *S. aureus*. Sarmand et al. found that almost 80% of patients showed new bacterial growth after ELVR. Of these, *S. aureus* and *P. aeruginosa* were detected most often^36^. Interestingly, the same species were also found on colonized airway stents^37^. *P. aeruginosa* is also frequently detected in COPD patients, colonizing airways. Its presence is associated with recurrent exacerbations and higher mortality^34,38^. Prior to EBV implantation, microbiological cultures showed a variety of bacterial species. For instance, patient 10, who had a biofilm predominantly consisting of *S. aureus*, showed growth in the bronchial wash fluid even before implantation. This suggests that pre-existing bacterial colonization of the airways could be a source for biofilm formation on the EBVs. The finding of oral flora on EBV (Fig. 3) could be explained by a de-novo infection of EBV with bacteria from the oral cavity, which could be transferred into the lower airways by bronchoscopic procedures or aspiration. Thus, the source of bacteria for biofilm formation could be both pre-existing airway colonization and new pathological colonization post-procedure.

Other potential factors that might influence bacterial biofilm formation in patients with ELVR include the immune status of the patient, the number of EBVs each patient carries, the duration of implantation, and multiple EBV implantations. On average, the duration from EBV implantation to removal and FISHseq analysis was nearly a year (mean of 362.0 ± 352.4 days) for patients with active biofilm. While our findings suggest that a certain time is necessary for biofilm development, this is only a supposition as our pilot study did not systematically analyze every removed EBV for biofilm presence. Re-implantation of new EBVs after removal was performed in 3/5 of patients with biofilms. Although the exact impact of the number of EBV implantations on clinical outcomes is not fully understood, the presence of biofilm may necessitate more frequent interventions and replacements due to complications or lack of clinical benefit from the initial implantation. Additionally, the patient’s immune system might play a key role in EBV colonization and biofilm formation, allowing the growth of commensal bacteria in some individuals. Interestingly, we found that most patients did not have significantly elevated inflammatory markers at either the time of EBV implantation or removal. Biofilms are common in both acute and chronic lung infections, inducing an inflammatory response marked by IL-1β and TNF-α^39^. These markers indicate ongoing inflammation, but not at levels seen in acute systemic infections, explaining the low systemic inflammatory markers in our patients. Inflammatory cells localize near bacteria within biofilms, showing that while the immune system can identify pathogens, it struggles to eradicate them due to the protective biofilm structure^39^. This immune evasion is facilitated by the biofilm matrix, which protects bacteria from phagocytosis^17,18^.

When we aimed to find clinical clues for the presence of biofilms, we found that macroscopic evidence of mucus layers on EBVs by CT or bronchoscopy was not exclusively associated with biofilm formation.

Over the recent years studies showed that a careful selection of patients improved the outcome of ELVR^40^. For instance, anatomic conditions such as fissure integrity as surrogate for absence of collateral ventilation turned out to be crucial for therapy success^41–44^. Commonly, patients with bronchitis phenotyp or a relevant immunsupression are almost not treated with EBV to avoid severe local microbiologic colonization^40^. Furthermore, it is recommend to begin prophylactic antibiotic therapy after ELVR^3^. As there is a high prevalence of positive microbiological cultures in patients with COPD undergoing ELVR^45^, and patients with colonized airways after ELVR tend to have more exacerbations, antibiotic therapy should given even prior to ELVR^3,20^. However, these recommendations are based on little evidence so far.

Our study provides some evidence that biofilm infection might influence the lack of clinical benefits, symptom worsening, and increased exacerbations leading to the decision of EBV removal in patients with ELVR. Patients with evidence of biofilm had poorer lung function at the 3-months follow-up compared to those without biofilm. Interestingly, this poorer lung function was not reflected in a subjective increase in symptom burden measured by CAT and SGRQ within this patient group. However, exacerbations within six months post-EBV implantation were more frequent in patients with biofilms. While the study provides evidence of biofilm infection on EBV for the first time in patients with recurrent exacerbations and EBV removal, the design of our study and small sample size have limitations in demonstrating the direct correlations between biofilms and clinical outcomes. Future studies, which address the correlation of treatment strategies/outcome and bacterial colonization/biofilm formation during ELVR, are needed. FISHseq could here enhance microbiological diagnostics for patients with ELVR, aiding in future therapy decisions regarding EBV implantations.

This pilot study has several limitations, including the absence of a control group and the reliance on retrospective clinical data collection. Only patients with recurrent exacerbations or severe pneumonia who underwent EBV removal were included, while asymptomatic patients were not analyzed. This exclusion may result in selection bias. However, identifying a suitable control group presented significant challenges. Additionally, there is a lack of standardization in treatment protocols. In clinical practice, EBVs are typically not removed from asymptomatic patients, which limits the ability to investigate outcomes in this subgroup. The study focused on patients with severe complications, potentially skewing the findings. Including asymptomatic patients or those with a range of outcomes would provide a more comprehensive understanding of the impact of biofilms on EBV in patients with ELVR. However, such a study design was not feasible at this time. Furthermore, the inclusion of only 10 patients restricts the statistical power and generalizability of our findings. As the first exploration of biofilms in patients with ELVR, our results should be considered preliminary. Thus, this highlights the need for further research to confirm our observations. To address these limitations, a larger, prospective study with increased patient numbers and adequate funding should be conducted to validate our findings and enhance their applicability to a broader patient population. In a potential prospective study, patients could be systematically assessed at baseline and during regular follow-up visits, which would include detailed clinical evaluations and the collection of microbiological samples for microbiome profiling. All removed valves should be regularly examined for biofilms using FISHseq and correlated with clinical and microbiological data. Additionally, advanced techniques such as single-cell analysis could be used to delve deeper into the inflammatory response associated with biofilm infections. This method would help to understand the specific cellular responses and interactions occurring within the biofilm microenvironment.

Taken together our findings demonstrade bacterial biofilms on EBV for the first time using the advanced molecular microscopic method FISHseq. Patients with biofilm infection on EBV exhibited increased exacerbations and clinical worserning, often leading to the decision of EBV removal. FISHseq may contribute to EBV diagnostics, helping to optimize treatment and prevent failure in patients undergoing ELVR. However, further prospective studies are needed to evaluate the clinical relevance of biofilm formation on EBV and appropriate treatment options to avoid infections in patients with ELVR.

## Electronic supplementary material

Below is the link to the electronic supplementary material.

Supplementary Material 1

## Data Availability

All relevant data of patients analysed during this study is presented as supplementary information. Additional data are available from the corresponding author upon reasonable request.

## Abbreviations

COPD: Chronic obstructive pulmonary disease
ELVR: Endoscopic lung volume reduction
EBV: Endobronchial valves
FISH: Fluorescence in situ hybridization
FISHseq: FISH + PCR/sequencing
