# Supplementary material for: Biofilm infections of endobronchial valves in COPD patients after endoscopic lung volume reduction: a pilot study with FISHseq
Source: Sci Rep. 2024 Oct 4;14:23078. doi: 10.1038/s41598-024-73950-3 (PMC11452729; doi:10.1038/s41598-024-73950-3)
Supplement: Supplementary file 1 — Supplementary Material 1 [file 41598_2024_73950_MOESM1_ESM.docx]

**Supplement Table 1: Detailed patient informations.**

| **Patient ID** | **1** | **2** | **3** | **4** | **5** | **6** | **7** | **8** | **9** | **10** |
| --- | --- | --- | --- | --- | --- | --- | --- | --- | --- | --- |
| **FISHseq Proof of active Biofilm** | yes | yes | yes | no | no | no | yes | no | no | yes |
| **Gender** | f | m | f | m | f | f | f | f | m | f |
| **Age (y)** | 61 | 74 | 58 | 78 | 60 | 67 | 59 | 68 | 62 | 66 |
| **Pack Years** | 80 | 50 | 45 | 40 | 40 | 50 | 30 | 60 | 80 | 45 |
| **GOLD Classification** | GOLD III | GOLD IV | GOLD IV | GOLD III | GOLD IV | GOLD III | GOLD IV | GOLD III | GOLD IV | GOLD IV |
| **Lung function at baseline** |  |  |  |  |  |  |  |  |  |  |
| FEV1 (L) | 0.73 | 0.71 | 0.45 | 0.89 | 0.5 | 0.81 | 0.57 | 0.91 | 0.59 | 0.5 |
| FEV1 (%) | 31 | 25 | 19 | 34 | 26.3 | 39 | 24 | 42 | 21 | 21 |
| RV (L) | 5.08 | - | 6.22 | 6.78 | 3.65 | 3.62 | 5.91 | 4.18 | 5.1 | 5.06 |
| RV (%) | 253 | 188 | 334 | 248 | 213.4 | 185 | 274 | 203 | 215 | 243 |
| DLCO (mmHg) | 2.18 | - | 1.57 | 3.1 | 1.59 | 2.86 | 1.03 | 2.93 | 3.24 | 1.13 |
| DLCO (%) | 27 | - | 20 | 39 | - | 40 | 13 | 39 | 40 | 15 |
| pCO2 (mmHg) | 39 | - | 38 | 38.7 | 44.6 | 37.9 | - | 32.7 | 31.7 | 36.7 |
| **6-MWD (m) baseline** | 375 | - | 140 | 340 | - | 115 | 55 | 270 | 170 | 280 |
| **CAT (points) baseline** | 19 | - | 29 | 28 | 32 | 30 | 38 | 31 | 19 | 29 |
| **SGRQ (points) baseline** | 41.89 | - | 77.7 | 73.53 | - | - | 95.2 | 77.63 | 53.78 | 75.75 |
| **mMRC (points) baseline** | 2 | - | - | - | 3 | 2 | 4 | 4 | 3 | 1 |
| **Lung function at 3-Month-Follow-up** |  |  |  |  |  |  |  |  |  |  |
| FEV1 (L) | 0.54 | 0.84 | 0.39 | 0.72 | - | 0.72 | 0.54 | 0.95 | 0.78 | 0.6 |
| FEV1 (%) | 24 | 30 | 17 | 27 | - | 35 | 23 | 44 | 25 | 25 |
| RV (L) | 5.24 | - | 6.9 | 7.30 | - | 3.67 | 1.3 | 3.69 | 6.2 | 4.4 |
| RV (%) | 259 | 191 | 368 | 200 | - | 185 | 123 | 178 | 261 | 210 |
| DLCO (mmHg) | 1.65 | - | 1.96 | 3.86 | - | 2.39 | 1.71 | 3.72 | 2.02 | 2.05 |
| DLCO (%) | 21 | - | 25 | 48 | - | 34 | 23 | 48 | 23 | 25 |
| **pCO2 (mmHg)** | 40.6 | - | 37.7 | 41.6 | - | 36.9 | - | 40.1 | 38.7 | 36.3 |
| **6-MWD (m) 3-Month-Follow-up** | 225 | - | 140 | 360 | - | 190 | 350 | 280 | 365 | - |
| **CAT (points) 3-Month-Follow-up** | 21 | 16 | - | - | - | 32 | 14 | 27 | 25 | - |
| **SGRQ (points) 3-Month-Follow-up** | 44.09 | - | - | 70 | - | 65.14 | 43.59 | 78.31 | 42.14 | - |
| **mMRC (points) 3-Month-Follow-up** | 2 | 3 | - | - | - | 3 | 3 | 3 | 1 | - |
| **Comorbidities** | arterial hypertension | arterial hypertension,  chronic kidney failure, benign prostatic hyperplasia | arterial hypertension,  cachexia | coronary heart disease, coronary artery bypass grafting | deep vein thrombosis, hepatitis B | arterial hypertension,  myocarditis | heart failure, restless leg syndrome, cachexia | arterial hypertension, secondary adrenal insufficiency  cushing syndrome, hypo-thyroidism,  melanoma | pulmonary hypertension,  arterial hypertension,  AV-nodal reentrant tachycardia | osteoporosis,  vertebral pain syndrome, cachexia |
| **EBV**  **Implantation** | 04/2014: 3 EBV in RML  07/2014: 3 EBV in RML  04/2016: 1 EBV in RML  01/2018: 1 EBV in RML  08/2018: 3 EBV in RML | 09/2015: 6 EBV in LLL  01/2016: 1 EBV in LLL  05/2016: 3 EBV in LLL | 07/2015: 4 EBV in LUL  01/2016: 2 EBV in LML | 09/2015: 5 EBV in LLL | 10/2015: 5 EBV in LUL 03/2016: 3 EBV in LUL | 02/2018: 4 EBV in RUL and RML  08/2018:  2 EBV in RML | 11/2017: 3 EBV in RLL  08/2018: 4 EBV in RLL  03/2019: 7 EBV in LLL  02/2021: 1 EBV in LLL | 03/2017: 5 EBV in LLL | 08/2017: 3 EBV in LUL  01/2018: 2 EBV in LUL | 02/2020: 5 EBV in LLL 07/2020: 6 EBV in LLL |
| **EBV Removal** | **03/2016: 3 EBV in RML*** 10/2017: 1 EBV in RML  05/2018: 1 EBV in RML 11/2021: 2 EBV in RML | 01/2016: 1 EBV in LLL  **03/2016: 1 EBV in LLL***  10/2016: all EBV in LLL | 09/2015: 1 EBV in LUL **04/2016: all EBV in LUL*** | 02/2016: 4 EBV in LLL  **04/2016: 1 EBV in LLL*** | 02/2016: 3 EBV in LUL **08/2016: 5 EBV in LUL*** | **04/2018: 2 EBV in RUL*** | 05/2018: 2 EBV in RLL 01/2019: 5 EBV in RLL  **08/2021: 8 EBV in LLL*** | **02/2020: 1 in EBV*** | **08/2017: 1 EBV in LUL*** | **04/2020: 5 EBV in LLL*** |
| ***Date of EBV Removal** | 11.03.16 | 22.03.16 | 01.04.16 | 05.04.16 | 11.08.16 | 24.04.18 | 24.08.21 | 28.02.20 | 30.08.17 | 16.04.20 |
| **Time until EBV Removal/FISHseq Analysis** (d) | 603 | 182 | 115 | 203 | 247 | 62 | 860 | 1067 | 6 | 50 |
| **Documented reasons for EBV explantation** | Pneumonia, exacerbation | exacerbation, EBV dislocation | exacerbation, EBV dislocation | No clinical benefit | exacerbation | pneumonia,  exacerbation,  EBV dislocation | exacerbation | exacerbation, EBV dislocation | pneumo-thoraces | No clinical benefit |
| **Exacerbations** |  |  |  |  |  |  |  |  |  |  |
| Exacerbations within 6 Month after EBV Implantation (n) | - | 1 | 2 | - | 1 | 1 | 1 | 1 | - | 1 |
| Exacerbations within 6 Month after EBV Removal (n) | 1 | 1 | - | - | - | 2 | 1 | - | - | - |
| **Inflammatory marker**  (M ± SD) |  | | | | | | | | | |
| **At the time of valve implantation** *multiple time points in some patients |  | | | | | | | | | |
| White blood cells (3.9 - 10.5 x10^9^/L) | 8.72±3.1 | 9.7±0.8 | 12.9±3.6 | 10.6±0.0 | 8.7±10.7 | 7.0±0.0 | - | 9.5±0.0 | 12.4±0.0 | 12.0±0.0 |
| C-reactive protein (< 5mg/L) | 9.85±13.1 | 4.5±3.7 | 0.3±0.0 | 1.9±0.0 | 7.0±6.5 | 1.9±0.0 | - | 1.6±0.0 | 3.8±0.0 | 0.6±0.0 |
| **At the time of EBV removal** |  |  |  |  |  |  |  |  |  |  |
| White blood cells (3.9 - 10.5 x10^9^/L) | 6.57±0.0 | 12.4±0.0 | 10.69±0.0 | 10.2±0.0 | 10.2±0.0 | 13.2±0.0 | 11.7±0.0 | 11.7±0.0 | - | 14.2±0.0 |
| C-reactive protein (< 5mg/L) | 2.1±0.0 | 2.9±0.0 | 0.6±0.0 | 1.5±0.0 | 1.9±0.0 | 51.5±0.0 | 10.0±0.0 | 1.5±0.0 | - | 7.8±0.0 |
| **Radiology Findings of Mucus Layers after EBV Implantation** | Mucus layer | Mucus layer | No mucus layer | No mucus layer | No mucus layer | No mucus layer | Mucus layer | Mucus layer | No mucus layer | Mucus layer |
| **Atelectasis of target lobe** | yes | no | no | yes | no | yes | yes | yes | yes | No |
| **Bronchoscopic findings of mucus layers after EBV Implantation** | Mucus layer | Mucus layer | Mucus layer | Mucus layer | Mucus layer | Mucus layer | Mucus layer | Mucus layer | No mucus layer | No mucus layer |
| **Development of Granulation Tissue after EBV Implantation** | Granulation tissue | Granulation tissue | No granulation tissue | Granulation tissue | No granulation tissue | Granulation tissue | Granulation tissue | Granulation tissue | No granulation tissue | No granulation tissue |
| **Microbiology cultures of bronchial washing fluid** |  | | | | | | | | | |
| **Before ELVR** | *Escherichia coli, Klebsiella pneumoniae*  (both 07/2014) | --- | *oro-pharyngeal*  *bacterial flora (01/2016)* | *Haemophilus influenzae, Myco-*  *bacterium scrofulaceum,*  *Candida albicans*  (all 09/2015) | *oro-pharyngeal*  *bacterial flora (09/2015)* | *oro-pharyngeal*  *bacterial flora*  (11/2017) | --- | *oro-pharyngeal*  *bacterial flora* (01/2017) | *Aspergillus clavatus* | *S. aureus (11/2019), oro-pharyngeal*  *bacterial flora* (09/2019) |
| **After ELVR** | *P. aeruginosa (03/2016, 10/2017,05/2018, 08/2018), E. coli (03/2016, 10/2017, 05/2018), K. pneumoniae (10/2017, 05/2018),*  oro-pharyngeal  bacterial flora 03/2016, 10/2017, 05/2018) | *Serratia marcescens (01/2016)*, *K. pneumoniae (05/2016),*  oro-pharyngeal  bacterial flora (03/2016) | *S. maltophilia (04/2016),*  *oro-pharyngeal*  *bacterial flora (09/2016)* | *S. maltophilia,*   *Moraxella*  *branhamella catarrhalis,*  *oro-pharyngeal*  *bacterial flora*  (all 03/2020) | *Aspergillus fumigatus (02/2016, 03/2016), Proteus mirabilis (08/2016), S. aureus (08/2016), Acinetobacter baumannii (08/2016)* | *S. aures (04/2018, 08/2018, 02/2021), Penicillium* sp*. (08/2018),*  *S. marcescens (11/2021), oro-pharyngeal*  *bacterial flora (08/2018, 01/2020, 02/2021, 11/2021)* | *K. pneumoniae (01/2019, 08/2020, 08/2021),*  *S. aureus (01/2019, 08/2020, 08/2021),*  *oro-pharyngeal*  *bacterial flora (01/2019)* | *oro-pharyngeal*  *bacterial flora (01/2018, 10/2019, 10/2020, 10/2021), Aspergillus fumigatus (12/2018),*  *S. aureus (10/2019)* | *P. aeruginosa (01/2018)* | *oro-pharyngeal*  *bacterial flora (04/2020, 07/2020),*  *S. aureus (07/2020)* |
| **Antibiotic therapy during**  **intervention** | Prolonged antibiotic therapy | Prolonged antibiotic therapy | Prolonged antibiotic therapy | Prolonged antibiotic therapy | Prolonged antibiotic therapy | single shot antibiotic therapy | Prolonged antibiotic therapy | single shot antibiotic therapy | single shot antibiotic therapy | single shot antibiotic therapy |
| **Antibiotic therapies** | Ampicillin/  Sulbactam, Azithromycin, Piperacillin/  Tazobactam | Moxifloxacin | Ampicillin/  Sulbactam,  Clari-thromycin | Ampicillin/  Sulbactam | Moxifloxacin, Piperacillin/  Sulbactam | Piperacillin/  Tazobactam,  Levofloxacin,  Azithromycin | Piperacillin/  Tazobactam, Meropenem | Piperacillin/  Tazobactam, Ciprofloxacin, Ampicillin/  Sulbactam, Levofloxacin, Moxifloxacin | Piperacillin/  Tazobactam, Azithromycin, Meropenem | x |
|  |  |  |  |  |  |  |  |  |  |  |

*FISHseq =FISH + PCR/sequencing; FEV 1= Forced Expiratory Pressure in 1 Second; RV= Residual Volume; DLCO=Diffusion capacity;6-MWD=6-Minute Walk Test; CAT-Score=COPD Assessment Test; SGRQ=St. George’s Respiratory Questionnaire; mMRC-Score=Modified British Medical Research Council; EBV=Endobronchial Valves; CRP=C-reactive Protein; WBC=White blood; M ± SD =Mean, Standard Deviation; RML = Right Middle Lobe; RUL = Right Upper Lobe; RLL = Right Lower Lobe; LUL = Left Upper Lobe; LLL = Left Lower Lobe, M ± SD =Mean, Standard Deviation* **** Date of FISHseq Analysis***
